# Evaluation of stressors and coping strategies for stress in Indian anaesthesiologists

**DOI:** 10.4103/0019-5049.79871

**Published:** 2011

**Authors:** RV Shidhaye, DS Divekar, VK Dhulkhed, Gaurav Goel, Arunkumar Gupta, Rahul Shidhaye

**Affiliations:** Department of Anaesthesiology and Critical Care, Pravara Institute of Medical Sciences, Loni, India; 1 Indian Institute of Public Health, Hyderabad, India

**Keywords:** Anaesthesiologists, strategies to cope, stress, stressors

## Abstract

Several studies have been done to assess job satisfaction and quantify effects of stressors on anaesthesiologists in different regions and countries. Studies related to stress in Indian anaesthesiologists are very limited, which prompted us to design this study not only to identify the stressors but also to find out how anaesthesiologists react to stress and devise means to minimize it to increase their job satisfaction levels. A set of questions was handed over personally to 200 anaesthesiologists at the national- and state-level anaesthesiology conferences and continuing medical educations with a request to return them duly filled in, with an assurance that confidentiality and anonymity would bemaintained. Main outcome measures were demographics, factors causing stress, how the responding anaesthesiologists and their colleagues react to it and methods they adopt to reduce stress at their workplace. Response rate was 96%. The total number of respondents was 192 (54% males and 46% females; juniors, 76%; and seniors, 24%). Identified stressors were as follows: time constraints (34%), medicolegal concerns (24%), interference with home life (22%), clinical problems (20%) and communication problems (9%). Different strategies for coping with stress were identified. This survey is just a beginning. Indian Society of Anaesthesiologists is requested to look into the matter and take it further on a larger scale by multicentric studies to lay down standards related to number of working hours, number of night-call duties per week, proper assistance, medicolegal protection, etc., which would not only reduce occupational stress but also improve efficiency and job satisfaction among anaesthesiologists.

## INTRODUCTION

The scope of work of anaesthesiologists in hospital practice has now expanded to include emergency care, intensive care and management of acute and chronic pain. In addition, some anaesthesiologists indulge in research, teaching and/ or shoulder administrative responsibilities. Consequently demand-supply gap for anaesthesiologists has greatly increased, and they are overworked. Conflicting demands are regarded as a risk factor for overwork.[1] Kain and colleagues[2] reported that many anaesthesiologists exhibit symptoms of chronic stress. Sources of chronic stress include not only competence-related factors, production pressures, long working hours, night call and fatigue but also associated problems like fear of litigation, economic uncertainty and interpersonal relationships.[1,3–5] Lindfors *et al*.[6] found that 1 in 4 Finnish anaesthesiologists seriously thought of committing suicide some time or the other, with higher incidence in persons with poor health, low social support and family problems. Risk factors at work were conflicts with co-workers and superiors, on-call–related stress symptoms and low organizational justice. According to Lindfors *et al*.,[7] job control and organizational justice were found to successfully mitigate stress symptoms among those who had on-call hospital duties; and enhancing their decision-making procedures, interpersonal relations and job control routines was found to prevent on-call stress and related symptoms. Kazuyoshi Kawasaki *et al*.[8] showed work-related stress among anaesthesiologists is related to workload and other factors. Several studies have been conducted to find out job satisfaction and quantify effects of stressors among anaesthesiologists in different regions and countries.[9–16] Studies related to stress in Indian anaesthesiologists are very limited, which prompted us to design this study not only to identify the stressors but also to find out how anaesthesiologists react to stress and find ways to minimize them.

## METHODS

A set of questions was handed over personally to 200 anaesthesiologists at the national- and state-level anaesthesiology conferences and CMEs with a request to return them duly filled in, conveying that it would take a very short time to complete the questionnaire and with an assurance that confidentiality and anonymity would bemaintained. Questionnaire used by Kluger *et al*.[14] for assessing job satisfaction, stress and burnout in Australian specialist anaesthetists was used with necessary modifications. Respondents were allowed to choose one or more options.

National conference is attended by residents and senior anaesthesiologists from teaching as well as community hospitals from all over India. State-level conferences and CMEs were selected because many practicing anaesthesiologists from nonteaching private hospitals attend these.

Our sample size of 200 was based on previous similar studies found in literature.[3,10,13,17–19]

Participants were randomly selected. Every fourth person met and willing to participate was included.

Design:Confidential survey of various groups.

Subjects:Doctors undergoing residency trainingprogram in the specialty of anaesthesiology and qualified practicing specialist anaesthesiologists.

Main outcome measures:Demographics, factors causing stress, how responding anaesthesiologists and their colleagues react to it and methods they adopt to reduce stress at their workplace.

Anaesthesiologists having more than or equal to 8 years of practice were grouped as seniors; and those having less than 8 years of practice, as juniors. Professors and associate professors were included in the ‘seniors’ group, while residents and lecturers were included in the ‘juniors’ group.

Data analysis was done using STATA 10. Comparisons of categoricalvariables were performed using chi-square analysis. A Pvalue of <0.05 was considered statistically significant.

## RESULTS

Out of 200 questionnaires given to the practicing anaesthesiologists and postgraduate residents, 192 were returned (96% response rate). This high response rate was due to the fact that questionnaires were given and collected personally. All percentage figures are expressed in whole numbers, ignoring fractions for simplicity in discussion [Table 1].

**Table 1:** Demographics

	Age in years	Number of respondents (%)
Age-wise distribution	25 to 34	135 (70)
	35 to 44	36 (19)
	45 to 54	15 (8)
	55 to 64	4 (2)
	> 65	2 (1)
Gender-wise distribution	Males	104 (54)
	Females	88 (46)
Distribution according to the number of years in practice	0 to 4	117 (61)
	5 to 8	29 (15)
	9 to 12	18 (9)
	>12	28 (15)
Type of hospital where respondents work	Teaching hospital	124 (65)
	Community hospital	68 (35)
Distribution according to weekly working hours	Average working hours per week	
	<50	74 (38)
	51 to 60	57 (30)
	61 to 70	36 (19)
	71 to 80	23 (12)
	>80	2 (1)
Number of OTs in respondent’s hospital	1 to 4	75 (39)
	5 to 9	60 (31)
	10 to 14	23 (12)
	> 15	34 (18)

OTs: Operation theatres

The total number of juniors was 146 (76%), in which there were 79 males and 67 females. The total number of seniors was 46 (24%), which included 25 males and 21 females.

Factors making the job as an anaesthesiologist stressful were reported to be as follows:time constraints, according to 65 (34%) respondents; interference with home life, according to 42 (22%); medicolegal concerns, according to 46 (24%); communication problems, according to 17 (9%); and clinical problems, according to 39 (20%) respondents.

Anaesthesiologists reported reacting to stress in the following ways: 60 (31%) respondents discussed with colleagues; 74 (39%) discussed with partner; 46 (24%) pursued non-medical activities; 30 (16%) used to become irritable; 13 (7%) travelled; 12 (6%) heightened concentration; 5 (2.5%) took to alcohol; 4 (2%) smoked; and 1 (0.5%) took drugs.

According to respondents, their colleagues reacted to stress in the following ways: 75 (39%) colleagues discussed with other colleagues; 15 (8%) ranted and raved; 35 (18%) used to become irritable; 14 (7%) travelled; 27 (14%) took leave; 17 (9%) took to alcohol; 23 (12%) smoked; and nobody took drugs.

To reduce stress at workplace, following suggestions were made by respondents: ‘Have experienced assistants’, according to 42 (22%) respondents; ‘Have better work organization’, according to 48 (25%); ‘Develop group cohesion’, according to 35 (18%); ‘Prioritize home-work commitments’, according to 17 (9%); ‘Find ways to control life’, according to 13 (7%); ‘Improve funding’, according to 10 (5%); and ‘Avoid solo practice’, according to 62 (32%) respondents [Table 2].

**Table 2:** Gender - and seniority-wise comparisons

Comparison in relation to	Males	Females
React to stress by being irritable	14	16*
Discuss with partner as a method to reduce stress	38	36*

	**Juniors**	**Seniors**

React to stress by being irritable	22	8*
Discuss with partner as a method to reduce stress.	52	22*
Choose to avoid solo practice as a method to reduce stress	38	24***

	**From teaching hospitals**	**From community hospitals**

Think better work organization is a method to reduce stress	25	23**
Choose to avoid solo practice as a method to reduce stress	32	30***

* >.05

** <.05

*** <.01

## DISCUSSION

Anaesthesiology has been previously identified as a stressful specialty.[2] Burnout, characterized by emotional exhaustion, depersonalization and lowered sense of professional accomplishment, is a consequence of chronic stress.[11] There are not many studies published on burnout in anaesthesiology even though this specialty is considered particularly stressful. Kain *et al*.[2] designed a study to track acute physiologic and behavioural processes in anaesthesiologists during occupation-specific stressful activities, such as induction of anaesthesia, and showed that it is associated with minor manifestations like rise in systolic as well as diastolic blood pressures and pulse rate. Many factors make practice of anaesthesiology stressful. On-call duty has been shown to be one of them.[7,9,20] During most emergencies which present at night times, reflexes of everyone working in the operation theatre are sluggish, and the assistants are tired due to insufficient rest and are preoccupied with preparations for the next day. To add to this, most emergency patients at night times are critical and need more vigilant attention. A survey that was conducted among retired members of the American Society of Anesthesiologists (ASA) has indicated that “demands of night call” is the most stressful aspect of anaesthesia practice, followed by “difficult” anaesthetic cases, liability issues, workload, burnout and economic issues.[3] Study by Hanlon *et al*.[20] suggested that sleepiness may be reduced by scheduling on-call duties to not more frequent than 1 in every 5 nights and by ensuring that residents sleep for more than 2 hours while on call. Kluger *et al*.[14] also showed that stressful aspects of anaesthesia included time constraints and interference with home life. Even in our study, we found ’time constraints’ to be the most common cause of stress. Indian anaesthesiologists face the same conflicting factors as those faced by Australian anaesthesiologists,[14] like pressures to get lists going on time, to produce a rapid turnover and/ or to travel between different hospitals for conducting cases of varied types. Compounding this situation is the present ‘same-day’ admission policy, due to which anaesthesiologists are often pressurized into arriving early for work, reviewing documents and examining patients who have never been seen previously, obtain consent and induce anaesthesia in a short time-frame and that too in the context of higher expectation of providing safe anaesthesia and fast recovery. Anaesthesiologists find time management as well as management of organizational factors more difficult and stressful than management of clinical problems.[14] In our study, 34% of respondents reported ‘time constraints’ as the most common reason for stress, whereas only 20% reported ‘clinical problems’ as the most common cause of stress.

There are many perioperative complications that may occur due to some unavoidable circumstances which may be beyond the control of the anaesthesiologist’s skill and knowledge; and when they occur in an ASA Grade I patient, medicolegal problems are almost inevitable. These worries are always at the back of the mind of an anaesthesiologist while performing his/ her routine tasks, making him/ her anxiety prone, which adds to stress significantly. Twenty-four percent of anaesthesiologists found medicolegal aspects as a cause of increasing stress.

On occasions, surgeries last longer than expected, and an anaesthesiologist’s entire schedule gets upset. He/ She has to curtail the time scheduled for family and is compelled to miss social gatherings and functions on many occasions. He/ She finds it difficult to devote sufficient time to children because of long duty hours. These factors interfere with family life and add to other factors which contribute to stress. Twenty-two percent of the respondents indicated this in our study.

Many patients are in ASA Grades III, IV and V and are critical. A few surgeries like cardiothoracic, paediatric surgeries and neurosurgery continue for long duration and need greater attention all throughout. Patients having medical problems like diabetes mellitus, hypertension, renal disorders, chronic obstructive pulmonary disorder (COPD), etc., and their associated complications necessitate eternal vigilance. Minor errors in judgment can cause disaster. These clinical problems lead to stress. In our study, 20% of the anaesthesiologists positively reflected that this factor significantly contributes to causation of stress. Only 9% of the respondents indicated communication-related problems as a stressor.

There are many stress-reducing strategies. Supportive work and social environments are important compensatory mechanisms for a stressful work life. Capacity and capability of individuals left alone and unsupported by friends and family to respond to periods of stress are very limited. Colleagues, friends and family members, especially the spouse of the clinician, can play a great role in allaying the levels of stress. Thirty-one percent of the respondents agreed to this and said that they discuss their problems with their own colleagues, who can appreciate their problems better as they are also sailing in the same boat.

Other ways to reduce stress are to join clubs, participate in sports, listen to music, watch movies, go to picnics, etc. Anaesthesiologists can participate in non-medical activities like those conducted by Rotary and Lions clubs. In our study, 24% of the anaesthesiologists said they pursued such non-medical activities.

A spouse is a friend, a philosopher and a guide who thoroughly understands your problems and shares them sympathetically. In our study, 39% of the respondents (38 males and 36 females) said that they discuss their problems with their partner. No gender-wise difference was found regarding this aspect (*P*>0.05) among both groups — seniors and juniors(*P*>0.05). Forty-eight percent of senior anaesthesiologists and 36% of juniors discussed problems with their partner to reduce stress. This difference between seniors and juniors could be an erroneous reflection of facts because a large number of juniors could still be single. It is expected that more seniors would discuss their problems with their spouses because their long association goes a long way in appreciating and better understanding each other’s problems. But our findings were not consistent with this expectation.

During surgery, some anaesthesiologists are very irritable, become angry, start shouting at colleagues and may even abuse subordinates. There was no difference between juniors and seniors in this respect (*P*>0.05). Kluger *et al*.[14] found Australianfemale anaesthetists to have higher stress levels than Australianmaleanaesthetists. Though Indian females are relatively more emotional, their capacity to withstand stress is also much more. Probably this was the reason why we did not find gender-related difference with respect to response to stress by being irritable. To relieve tension, a few anaesthesiologists who cannot cope up with stress opt for alcohol and smoking at times. Fortunately in our study, only 2% to 3% of the respondents opted for alcohol and smoking. Only 1 respondent confessed to consuming drugs.

Since this profession involves a lot of stressful work and tension, it is essential to find ways to reduce stress, which can be achieved by better work organization, having a time-bound schedule to the extent possible and developing a congenial friendly group so that there is sharing and distribution of workload as well as enhancing of the possibility of taking leave whenever needed. A cohesive surgical team wherein each member of the team foresees the requirement of the other and appreciates the problems of the other, is well placed to deliver the goods in the most efficient manner in the shortest possible time. This fact has been realized and reported by high-risk industries like airlines, offshore oil drilling, etc. Eighteen percent of the respondents were in favour of having ‘group cohesion’ as an important factor to reduce stress.

Group practice is not only more efficient but also provides more earning than solo practice, and this fact is better understood and appreciated after having worked single-handedly under stressful conditions for a long period. Especially those working in small community hospitals would appreciate this better as most of them are private practitioners working single-handedly with very less sharing of responsibility. As a corollary, this fact is better appreciated by senior anaesthesiologists than juniors and by those working in nonteaching community hospitals (*P*<0.01).

The Australian Anaesthetic Incident Monitoring Study (AIMS) has shown that quality of anaesthetic assistance is associated with both the development and resolution of critical incidents.[21] In 5,837 reports, inadequate assistance was shown to contribute in 187 cases, whilst skilled assistance in 808 cases minimized the incidents. Adequately trained anaesthesia assistants are considered essential for the safe conduct of anaesthesia in Australia. Though it is not legally mandatory in India, it is surely essential for achievement of better working conditions for anaesthesiologists. Twenty-two percent of the respondents were in favour of having experienced assistants as an important factor to reduce stress.

Better work organization helps in better time management and job control, which subsequently reduces stress. This fact is also better understood and appreciated by those working in nonteaching community hospitals, who definitely need better work organization. In our study, 34% from community hospitals as against 20% from teaching hospitals were in agreement with this fact (*P*<0.05).

Limitation of our study: Our sample size was relatively small and may not be representative of all anaesthesiologists from India as it does not include a large number of practicing anaesthesiologists who never attend any conference or CME or workshop. Also the number of senior anaesthesiologists was relatively small.

This survey is just a beginning. It is sincerely felt that before drawing final conclusions and making any recommendations, large multicentric studies need to be done. Better working conditions in terms of ensuring availability of ergonomically designed anaesthetic equipments at all places (small or big hospitals); better remuneration for work done; good assistance; and limiting the number of working hours, both elective and emergency; would go a long way in reducing stress in members of this vital supportive specialty, as anaesthesiology is neither a diagnostic nor a therapeutic specialty. None else than the Indian Society of Anaesthesiologistsis well placed to look into these critical issues, and it is felt that it would look into this matter seriously.

The main beneficiary of these studies and strategies would be our VIP — the PATIENT, that too a VERY ILL one.

## References

[1] Seeley HF (1996). The practice of anaesthesia—a stressor for the middle aged?. Anaesthesia.

[2] Kain ZN, Chan KM, Katz JD, Nigam A, Fleisher L, Dolev J (2002). Anaesthesiologists and acute perioperative stress: A cohort study. Anesth Analg.

[3] Simpson LA, Grant L (1991). Sources and magnitude of job stress among physicians. J Behav Med.

[4] Jackson SH (1999). The role of stress in anaesthetists’ health and wellbeing. Acta Anaesthesiol Scand.

[5] Gaba DM, Howard SK, Jump B (1994). Production pressure in the work environment. Anaesthesiology.

[6] Lindfors PM, Meretoja OA, Luukkonen RA, Elovainio MJ, Leino TJ (2009). Suicidality among Finnish anaesthesiologists. Acta Anaesthesiol Scand.

[7] Lindfors PM, Heponiemi T, Meretoja OA, Leino TJ, Elovainio MJ (2009). Mitigating on-call symptoms through organizational justice and job control: A cross-sectional study among Finnish anaesthesiologists. Acta Anaesthesiol Scand.

[8] Kawasaki K, Sekimoto M, Ishizaki T, Imanaka Y (2009). Work stress and workload of full-time anaesthesiologists in acute care hospitals in Japan. J Anesth.

[9] Lindfors PM, Nurmi KE, Meretoja OA, Luukkonen RA, Viljanen AM, Leino TJ (2006). On-call stress among Finnish anaesthetists. Anaesthesia.

[10] Palmer-Morales LY, Gómez-Vera A, Cabrera-Pivaral C, Prince-Velez R, Searcy-Bernal R (2005). Prevalence of Burnout syndrome among anaesthesiologists in Mexicali. Gac Med Mex.

[11] Fernández Torres B, Roldán Pérez LM, Guerra Vélez A, Roldán Rodríguez T, Gutiérrez Guillén A, De las Mulas Béjar M (2006). Prevalence of burnout among anaesthesiologists at Hospital Universitario Virgen Macarena de Sevilla. Rev Esp Anestesiol Reanim.

[12] Morais A, Maia P, Azevedo A, Amaral C, Tavares J (2006). Stress and burnout among Portuguese anaesthesiologists. Eur J Anaesthesiol.

[13] Kluger MT, Bryant J (2008). Job satisfaction, stress and burnout in anaesthetic technicians in New Zealand. Anaesth Intensive Care.

[14] Kluger MT, Townend K, Laidlaw T (2003). Job satisfaction, stress and burnout in Australian specialist anaesthetists. Anaesthesia.

[15] Jenkins K, Wong D (2001). A survey of professional satisfaction among Canadian Anaesthesiologists. Can J Anaesth.

[16] Dai WJ, Chao YF, Kuo CJ, Liang KM, Chen TL (2009). Analysis of manpower and career characteristics of nurse Anaesthetists in Taiwan: Results of a cross-sectional survey of 113 institutes. Acta Anaesthesiol Taiwan.

[17] Mitra S, Sinha PK, Gombar KK, Basu D (2003). Comparison of temperament and character profiles of anesthesiologists and surgeons: A preliminary study. Indian J Med Sci.

[18] Kinzl JF, Knotzer H, Traweger C, Lederer W, Heidegger T, Benzer A (2005). Influence of working conditions on job satisfaction in anaesthetists. Br J Anaesth.

[19] Kinzl JF, Traweger C, Trefalt E, Riccabona U, Lederer W (2007). Work stress and gender-dependent coping strategies in anesthesiologists at a university hospital. J Clin Anesth.

[20] Hanlon JG, Hayter MA, Bould MD, Joo HS, Naik VN (2009). Perceived sleepiness in Canadian Anaesthesia residents: A national survey. Can J Anaesth.

[21] Kluger MT, Bukofzer M, Bullock M (1999). Anaesthetic assistants: Their role in the development and resolution of anaesthetic incidents. Anaesth Intensive Care.

